# Using dual-network-analyser for communities detecting in dual networks

**DOI:** 10.1186/s12859-022-04564-7

**Published:** 2022-01-10

**Authors:** Pietro Hiram Guzzi, Giuseppe Tradigo, Pierangelo Veltri

**Affiliations:** 1 Department of Surgical and Medical Sciences, Magna Graecia University, 88100 Catanzaro, Italy; 2 eCampus University, Novedrate, CO Italy

**Keywords:** Dual networks, Graphs, Densest subgraph, Communities, Social Networks

## Abstract

**Background:**

Representations of the relationships among data using networks are widely used in several research fields such as computational biology, medical informatics and social network mining. Recently, complex networks have been introduced to better capture the insights of the modelled scenarios. Among others, dual networks (DNs) consist of mapping information as pairs of networks containing the same set of nodes but with different edges: one, called physical network, has unweighted edges, while the other, called conceptual network, has weighted edges.

**Results:**

We focus on DNs and we propose a tool to find common subgraphs (aka communities) in DNs with particular properties. The tool, called Dual-Network-Analyser, is based on the identification of communities that induce optimal modular subgraphs in the conceptual network and connected subgraphs in the physical one. It includes the Louvain algorithm applied to the considered case. The *Dual-Network-Analyser* can be used to study DNs, to find common modular communities. We report results on using the tool to identify communities on synthetic DNs as well as real cases in social networks and biological data.

**Conclusion:**

The proposed method has been tested by using synthetic and biological networks. Results demonstrate that it is well able to detect meaningful information from DNs.

## Background

Network-based models have been widely used as a problem-solving strategy to analyse data interactions and relations in many domains. For example, in computational biology, network-based models are used to study relationships between biological macromolecules, and their associations [1–4]. In medicine, networks have been used to study patients [5, 6] and to model possible similarities among their conditions (e.g. co-morbidities). Even social network data can be modelled with graphs and analysed to extract relevant information regarding connections (e.g., similarities, shared interests) among users [7].

**Fig. 1:**
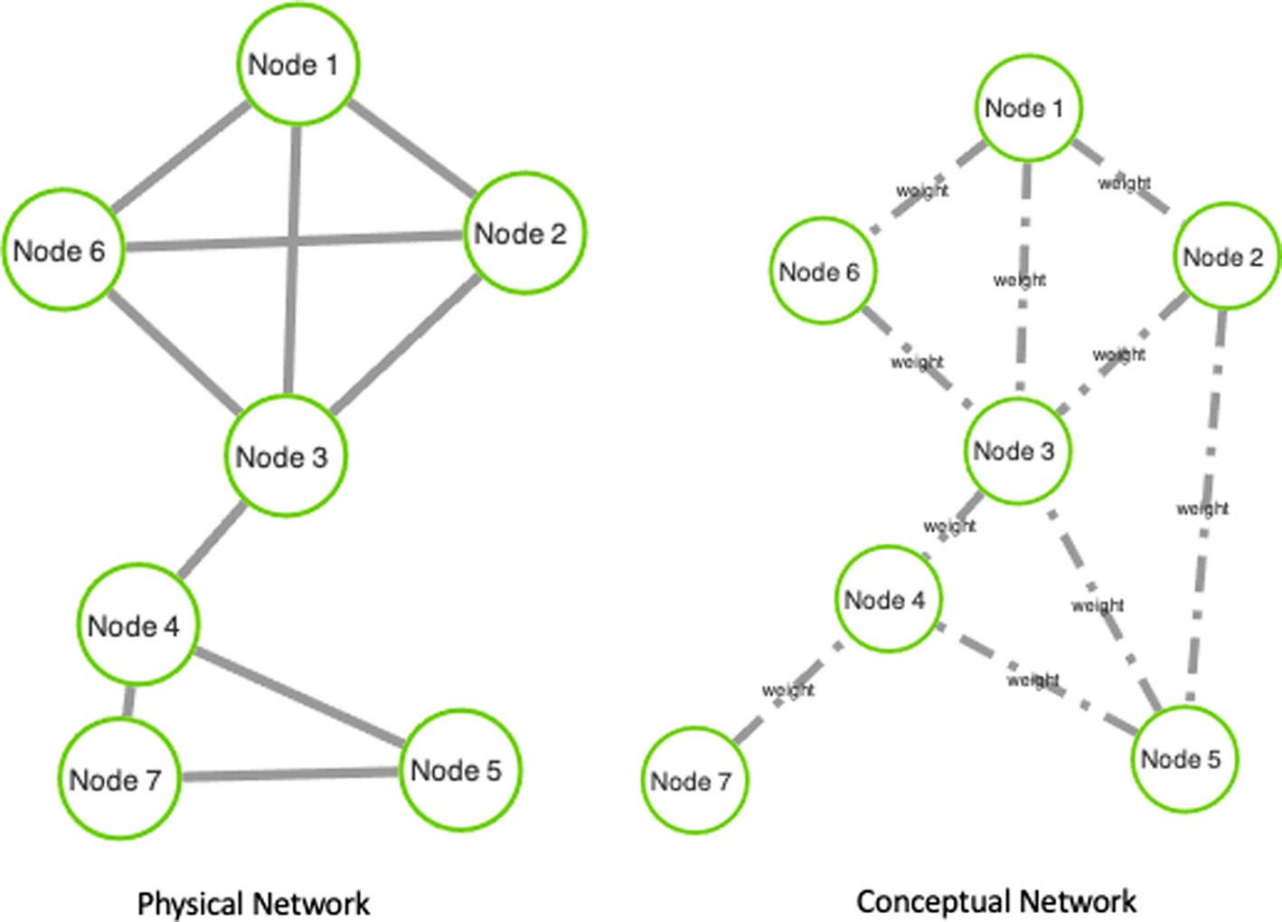
An example of dual network. The graph on the right (with dashed edges) represents the conceptual network, while the other one represents the physical network

**Fig. 2:**
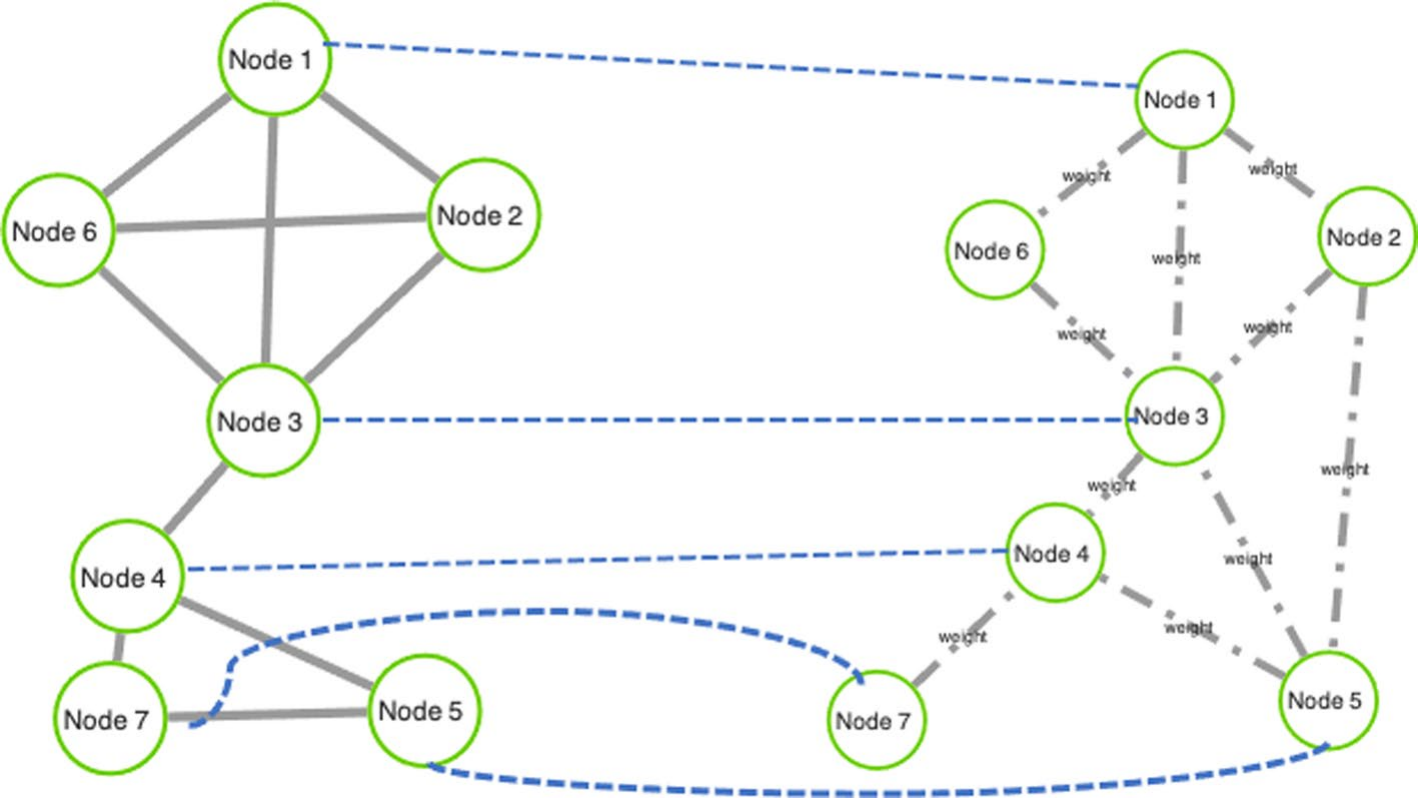
Figure shows an example of a dual network and relationship among nodes of physical and conceptual networks

**Fig. 3:**
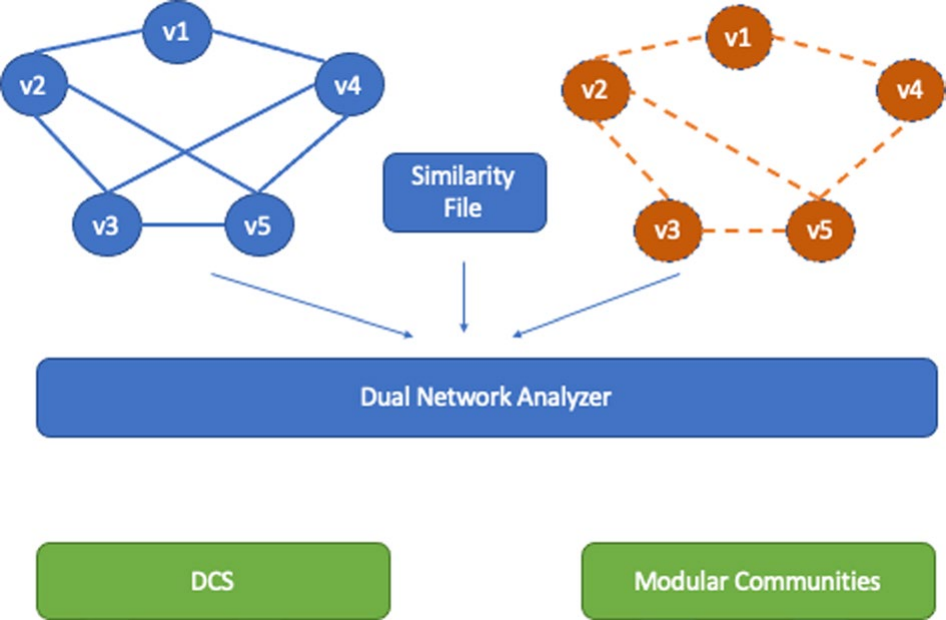
Workflow of the Proposed Algorithm. The algorithm receives as input two input networks (representing a dual network) and a list of nodes that should be mapped. Networks are initially merged together into a single Weighted Alignment Graph. Each node of the alignment graph represents a pair of nodes of the input network. Edges are inserted considering the two input networks. The Louvain algorithm is used for finding them modular communities, while in the case of DCS, then the Charikar algorithm is used. Each extracted sub-graph of the alignment graph represents a connected sub-graph of the unweighted networks and a subgraph of the conceptual network with the given properties (density or modularity)

**Fig. 4:**
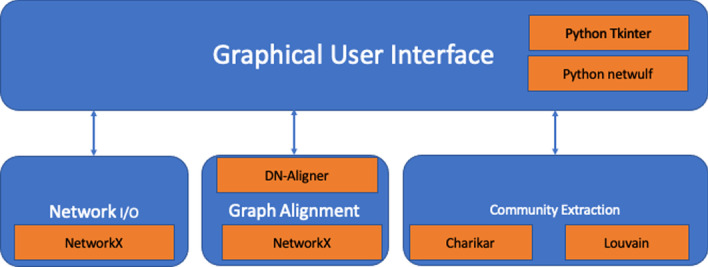
Architecture of the Dual Network Analyser tool

**Fig. 5:**
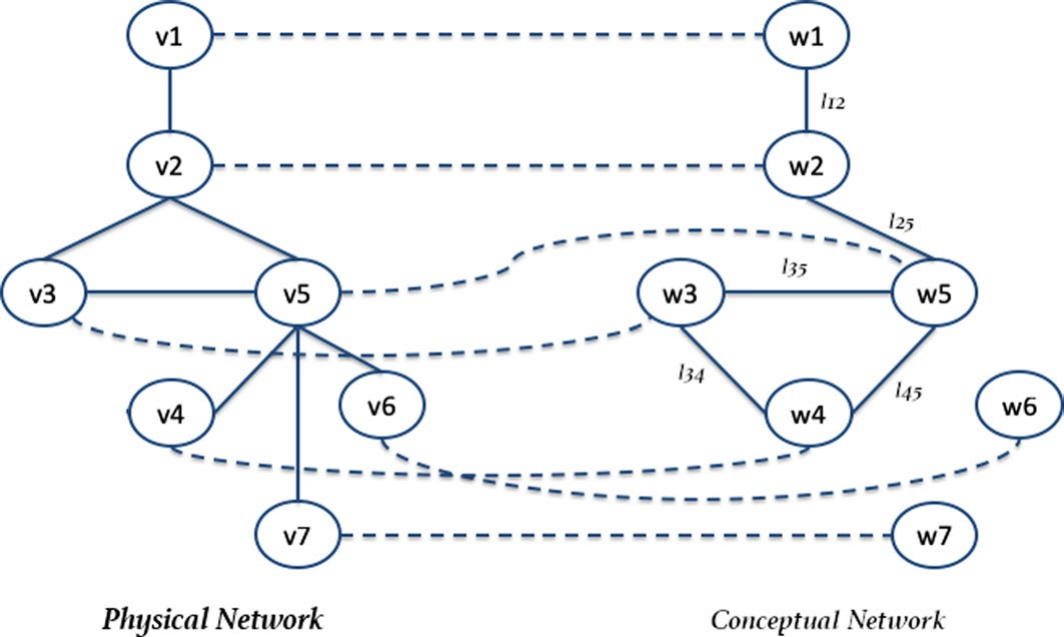
Alignment Example: the Algorithm receives as input two networks and a set of similarity relationship among nodes of the networks (dashed lines) [18]

**Fig. 6:**
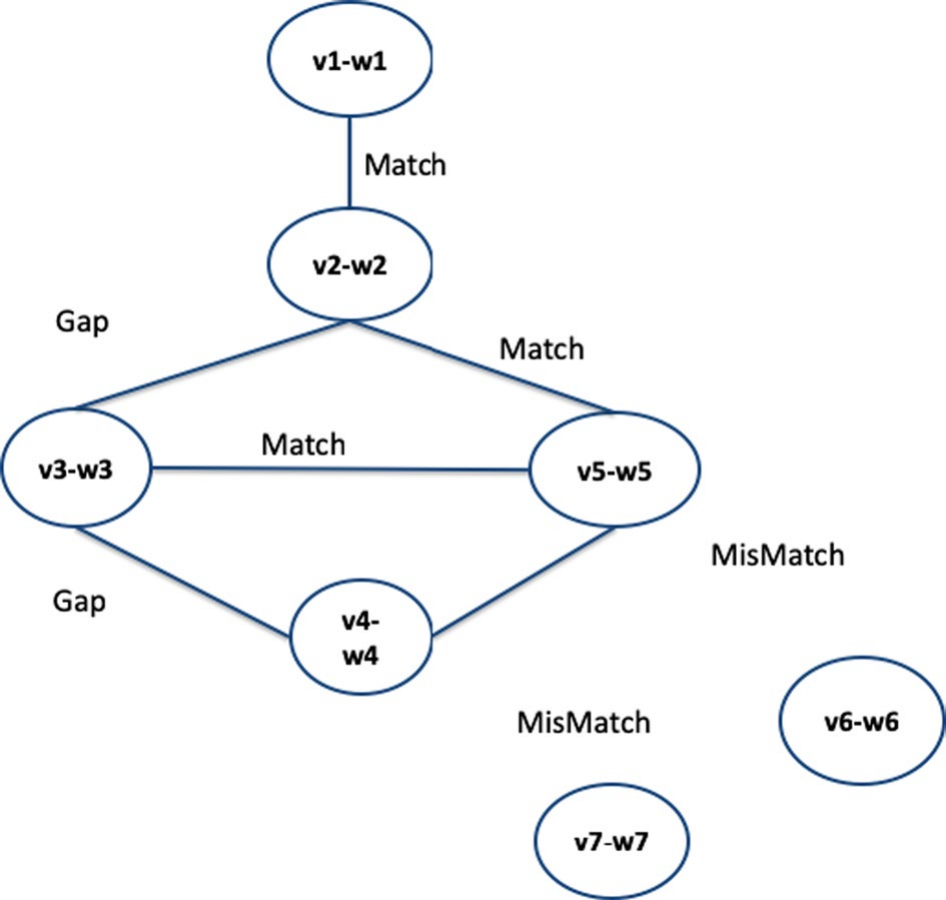
First, the algorithm builds the nodes of the heterogeneous alignment graph. The edges are then added according to the analysis of input networks

**Fig. 7:**
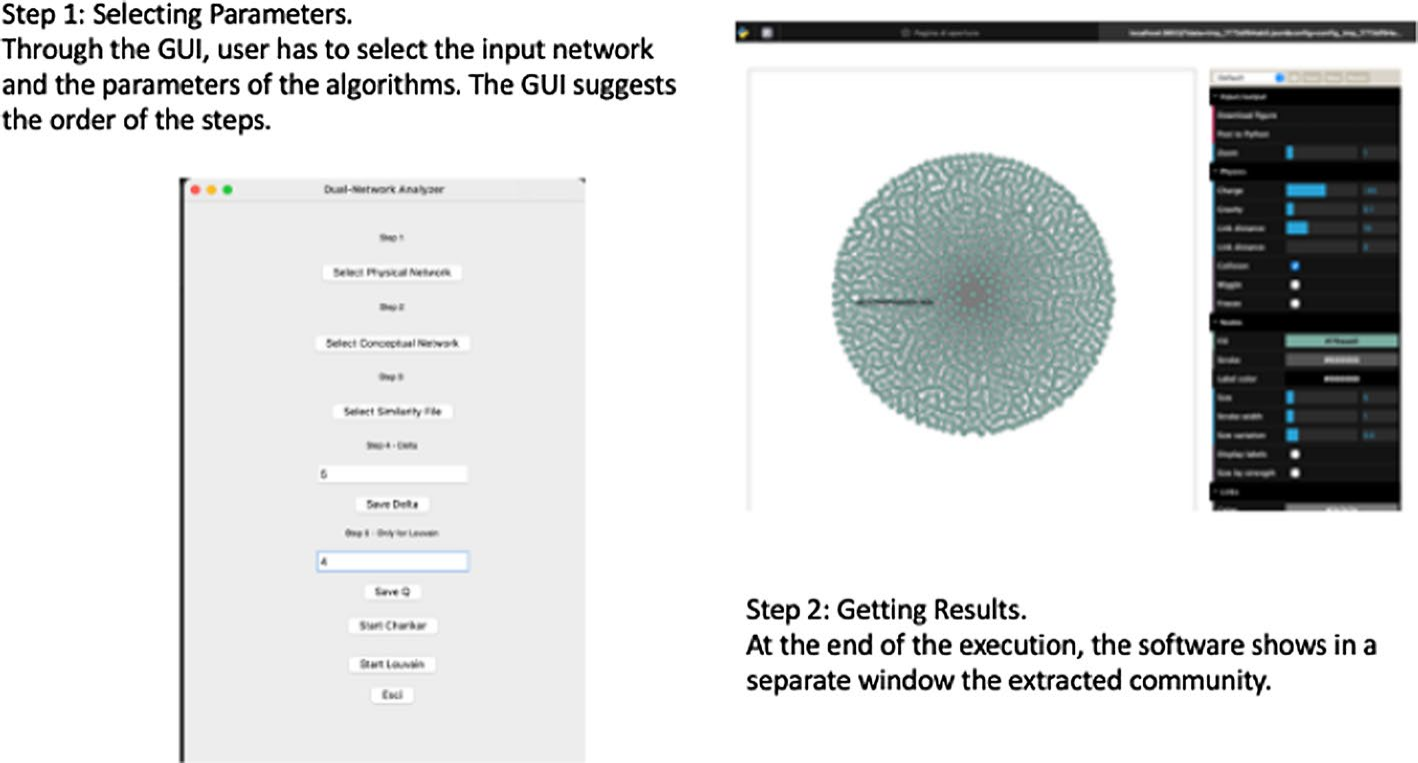
Graphical User Interface of the software tool

**Fig. 8:**
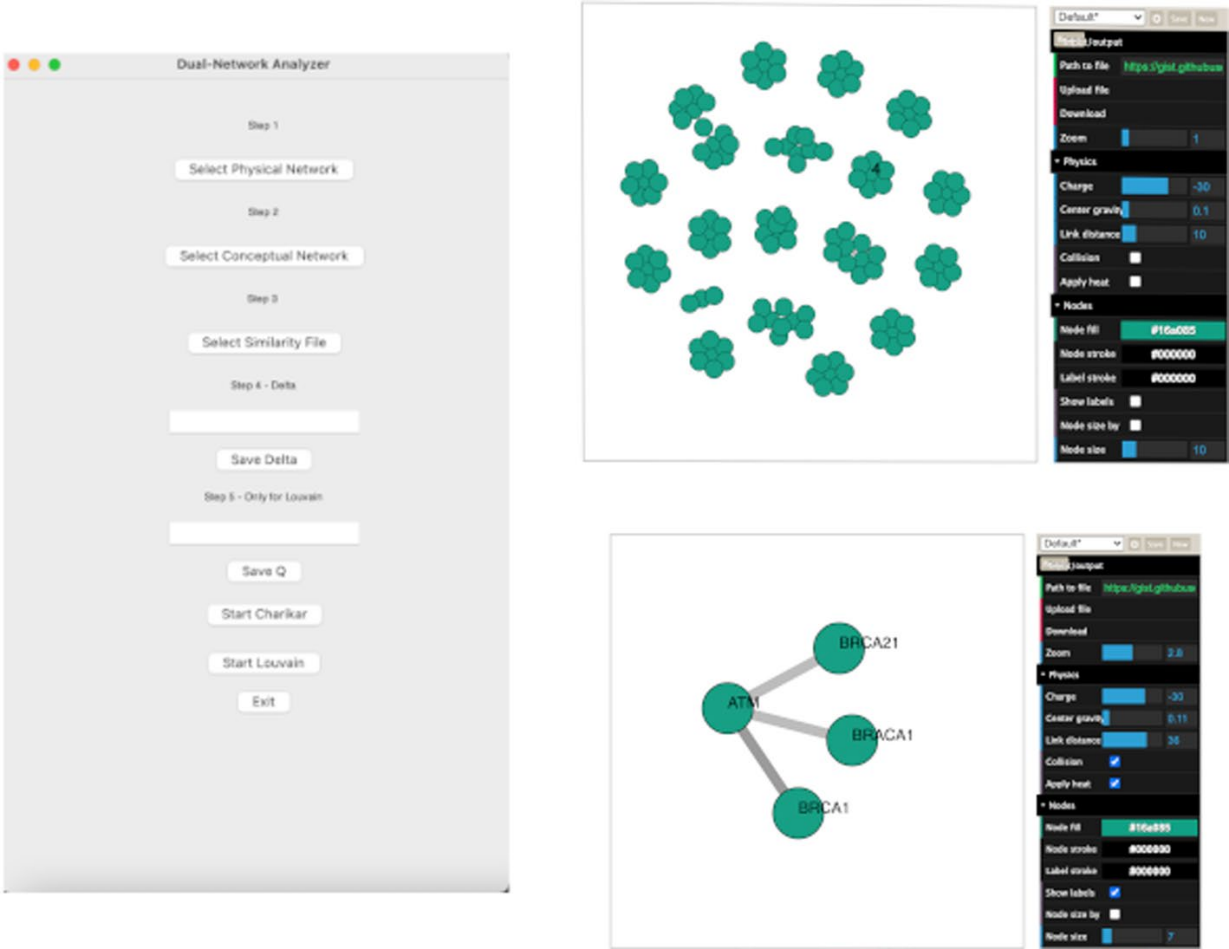
Figure depicts the use of DN-Analyser for the analysis of a biological network

**Table 1:** Execution times in milliseconds

Experiment	$T_{load}$ (ms)	$T_{align}$ (ms)	$T_{dcs}$ (ms)	$T_{louv}$ (ms)
1	10	100	150	200
2	15	200	250	300
3	21	300	350	400
4	23	500	450	500
5	35	1900	650	700

We performed the experiments on a MacOs MacBook Pro equipped with a 2.8 GHz Intel Core i7 quad-core and 16 Gb or Ram

**Table 2:** Number of nodes and edges for physical and conceptual networks used to test the Dual Network Tool

Experiment id number	Physical network		Conceptual network	
	Nodes	Edges	Nodes	Edges
1	500	1000	500	1500
2	1000	1500	1000	2000
3	1500	2000	1500	2500
4	2000	2500	2000	3000
5	2500	3000	2500	3500

Each line identifies the experiment number (i.e., 1 to 5)

**Table 3:** Performances on synthetic networks (average values are reported with their standard deviation). We evaluate the Mutual Information (MI) and the Rand index. We report MI and Rand as the average of all the found communities. We finally report the F-Score as the geometric average of MI and Rand, i.e. = F-Score = $$(2*Precision*Recall /(Precision+Recall)$$

Algorithm	MI	Rand	F-score
DN-analyser	0.71 ± 0.03	0.79 ± 0.02	0.73 ± 0.02
LOUVAIN	0.69 ± 0.04	0.68 ± 0.01	0.68 ± 0.02

**Table 4:** Performances on synthetic networks for average values of Positive Predictive Value (PPV), Sensitivity (SSN) and Accuracy (ACC) are reported with their standard deviation)

Algorithm	PPV	SSN	ACC
DN-Analyser	0.70 ± 0.01	0.78 ± 0.02	0.73 ± 0.02
LOUVAIN	0.68 ± 0.02	0.68 ± 0.01	0.68 ± 0.02

A considerable number of modelling approaches are based on the use of a single network (i.e., single set of nodes and edges) to represent data and the subsequent investigation of networks properties, such as community-related structures [8–11]. In computational biology, protein molecules and their biochemical associations are modelled using the Protein Interaction Network (PIN) formalism. In a PIN, communities represent protein complexes, i.e., a set of proteins bound together to play a specific role [12].

More recently, advanced models such as multilayer and multiplex networks have been proposed to model biological and social network data [13]. Also, to capture important attributes and to improve the mapping of real problems, a multiplex network model variant, called dual network (DN), has been defined. Where there is the need to model and study evolving phenomena [14, 15], graph pairs can be used to represent two different views of the same dataset.

A DN is a special case of multiplex networks in which only two layers are considered. Nevertheless, some differences should be taken into account. Multiplex networks have a given, fixed set of inter-layer edges and a set of intra-layer ones. Here we focus on the use of DNs in which: (*i*) one of the graphs is unweighted and referred to as a physical graph; (*ii*) the other one is edge-weighted and a called conceptual graph. The two graphs may have different (but overlapping) node sets; however, in many applications the nodes of the two graphs coincide. Figure 1 reports an example of DN. DNs are used to model two different types of relationships existing between nodes, which cannot be modelled with a single graph [16, 17]. For instance DNs are used when modelling physical and conceptual interactions, i.e. mapping two kinds of relations [18–23].

Adopting a DN to model real scenarios allows us to study interesting network properties using graph theory algorithms. For example, a Densest Connected Subgraph (DCS) [14] and [18]) may represent a set of related users of a social network, not necessarily connected. In a recommender system, a Densest Connected Subgraph (DCS) in a DN represents a set of nodes closely related to the conceptual network and connected to the physical one [14]. Similarly, to model (sub)sets of related genes and proteins, a *common modular graph* can be used to represent the subgraph having maximum modularity in the conceptual network and forming a connected component in the physical one.

In a network, finding a common modular graph is an NP-hard problem [14, 18, 24] in its general formulation. Techniques exist to solve the problem based on reducing it to the set cover problem [24] and others based on heuristics.

By using results reported in [18], and studying the application of the Louvain algorithm [25] on DNs, we present a novel graphical tool for finding common modular subgraphs in DNs. The tool also includes the Charikar algorithm starting from the results in [18]. The methodology is based on the following: a common modular subgraph is a set of nodes that induces a connected subgraph in the physical network and a subgraph with optimal modularity in the conceptual network. The proposed method receives as input: (*i*) the physical and conceptual networks, (*ii*) a set of correspondences among their nodes (see Fig. 2 for an example) and (*iii*) a set of parameters required for the process, which will be explained below. It is based mainly on two steps, as depicted in Fig. 3: (*i*) the two input networks are merged into a single weighted graph called *alignment graph*; and (*ii*) it uses the Louvain algorithm [25] for detecting the modular communities. The Louvain method is a greedy optimisation algorithm performing with large graphs by optimising modularity. The tool also allows us to find the densest communities by using the Charikar algorithm.

Each subgraph of the alignment graph induces a connected subgraph into the physical graph [18]. Moreover, while building the alignment graph, the weights of the conceptual graph are preserved. We use the edge weights as they are imported by the input data, and we do not consider edge weights as statistical indexes such as in [26]. Therefore, a subgraph of the alignment graph having maximum modularity induces both a connected subgraph in the physical graph and a subgraph with optimal modularity in the conceptual subgraph. The formulation of the problem we propose is based on a network alignment approach [9, 27–29]. We also consider the tuning techniques reported in [30, 31].

In the literature, there is a lack of analytic tools able for studying DNs. Hence, the proposed software prototype, named *Dual Network Analyser*, is able to support the user with the identification of modular communities from an input DN, as well as DCS identification. We show the effectiveness of our approach by presenting three case studies: (*i*) social network data, (*ii*) biological network data and (*iii*) synthetic network data.

### Related work

The analysis of communities with certain properties from an input graph (or network) is a recurrent problem in graph analysis research [17, 32–34]. We study DNs and focus on finding the Densest Connected Subgraph and the Modular Connected Subgraphs in a DN, which are both dense components of the considered graph. The detection of dense components in a graph has key applications in several fields, one of which is social network analysis [35–37]. Nevertheless, there are many definitions of graph *density*, which lead to the development of different algorithms. We believe that a correct definition of graph density is relevant to our problem. One definition of dense sub-graph is related to a fully connected sub-graph, also called *a clique*. However the identification of a maximal clique, also referred to as the *maximum clique problem*, belongs to the NP-hard complexity class [38], hence it is particularly difficult to approximate [39]. Wu et al. proposed an algorithm for finding the densest connected sub-graph in a DN [14] which uses a two-step strategy: first, it examines the DN and proceeds to prune it by eliminating nodes and edges that are not contained in the optimal solution; it then implements a greedy search strategy to find a DCS in the pruned DN. The approach implemented in Dual Network Analyser is more flexible. Indeed, (*i*) it allows greater flexibility in the DCS search, and (*ii*) it finds modular communities. Goldberg et al. proposed an algorithm based on the maximum-flow approach [40] to find the densest subgraph. Similarly, Asashiro et al. proposed a greedy algorithm based on the strategy of deleting the nodes of minimum degree [41]. Nevertheless, our heuristic method implements a similar approach, but we have added improvements by extending the method to support weighted graphs as well. There are also some variants of this problem, i.e. finding the top-*k* (overlapping) subgraphs of larger density [16, 42]. We also focus on the analysis of modular communities and on modelling phenomena and datasets which can gain clarity, expressiveness or significance when represented as DNs. In computational biology, dual networks have been used to represent co-expression of genes, and protein interactions in a unique framework [20]. In this formalism, authors built a weighted network representing co-expression among genes, (where the weight summarises the strength of the relation) and a physical network modelling the interactions of the corresponding determined proteins.

## Implementation

The developed tool, called the *Dual-Network-Analyser*, has been structured in modules, as represented in Fig. 4. The figure reports the main components (blue containers) of the tool, and the libraries (orange boxes) implemented and used to build the system. The main components functionalities can be summarised as follows:

- *Graphical User Interface*, which helps users to select and define parameters used during the execution of the algorithm. Based on the Tkinter library [43], it is also responsible for visualising graphs that are made possible by wrapping the Netwulf opensource library [44];
- *Network Input/Output*, is responsible for the reading networks from input files and for managing network representations during execution. It is also in charge of exporting results in files. This module is based on the open-source NetworkX library [45], a package able to create and manipulate networks efficiently;
- *Graph Alignment* is responsible for the alignment of the physical and conceptual networks. We wrapped and included libraries developed in [18] and available online^1^, which include the graph alignment algorithm and the Charikar algorithm implementations;
- *Community Detection* is responsible for detecting communities from the alignment graph. It is written by reimplementing the Charikar algorithm (available online at the above-cited codeocean URL), and includes the implementation of the Louvain Algorithm [25] of the cdlib Python Library^2^.

The graph alignment algorithm is based on two main steps: (*i*) building the alignment graph, and (*ii*) analysing the alignment graph. The first reuses the algorithm defined in previous work [18], and improved for Dual Network Analyser targets. It is responsible for the alignment of the physical and conceptual networks. We include previously developed libraries (see [18]) which are available online^3^. We shall now describe briefly the alignment algorithm. Let’s consider the following example: given two graphs, $G_1$ and $G_2$, where $G_1=(W,E_1)$ is a weighted graph (*conceptual network*) and $G_2=(V,E_2)$ is an unweighted one (*physical network*), let $f \subseteq V\times W$ be an initial set of correspondences between $G_1$ and $G_2$ nodes. We build a new graph *G* where each node is built by considering their associations with the conceptual graph $G_1$ and the physical one $G_2$. For instance, given a correspondence between nodes *v*1 and *w*1, there will be a new node in *G* named $(v1-w1)$. The conceptual and physical graph reported in the top part of Fig. 5 are mapped in the new graph reported in the bottom part. The two nodes *v*1 and *w*1 linked by a dashed line are used to build node $v1-w1$. Edges in graph *G* are built by considering the edges contained in the two input graphs. For instance, with regard to Fig. 5 there is an edge (*v*1, *v*2) between the *v*1 and *v*2 nodes, and an edge (*w*1, *w*2) between nodes *w*1 and *w*2, hence graph *G* will contain an edge between the two node $v1-w1$ and $v2-w2$. Since there is an edge in both the conceptual and the physical graph, the latter egde is marked as *Match*. Considering the nodes $v2-w2$ and $v3-w3$, since there is only one edge in the physical network (among nodes *v*2 and *v*3, the corresponding edge in *G* connecting $v2-w2$ and $v3-w3$, the edge is marked as *Gap*. Finally, in the case of missing edges among nodes in both the physical and conceptual graph, the nodes built in the alignement graph may not present any edge (e.g. see node $v7-w7$). All nodes are examined and after the analysis of all node pairs, the alignment graph is built, as represented in the bottom part of Fig. 6. The alignment procedure receives two networks, a file containing a set of relations between nodes and a threshold value $\delta$ mapping the connectivity constraints, and generates a weighted alignment graph which is included in the Dual Network Analyser tool, implemented by the Python language. The $\delta$ parameter is used by the algorithm to weight the relevant distance of the nodes. The user can tune such a value by also considering the dimension and structure of the input graphs.

The community detection module implements the Louvain algorithm to detect modular communities applied to a conceptual weighted network. This is used to evaluate communities that are connected in the physical network. For each detected community the module implementing Louvain, considers the corresponding induced subgraph on the physical network, and removes the nodes from the community until the induced subgraph is connected. The community detection module also includes the Charikar algorithm to detect the densest communities and users can choose between the two by using an implemented graphical user interface. This tool provides a Graphical User Interface (depicted in Fig. 7), which is based on *TKinter* Python library [43]. A graph visualisation module has been implemented by wrapping the *Netwulf* open source library [44–46], an interactive visualisation library that can efficiently create and manipulate *NetworkX* [45, 46] data structures.

## Results

We used and tested the Dual-Network-Analyser tool on DNs. Starting from DNs, the tool allows us to find the densest connected sub-graph (DCS), i.e. the one with the highest density in the conceptual network while also connected to the physical network. This was tested on the problem of finding communities in DNs. In order to run the experiment, the user interacts with the GUI depicted in Fig. 7 and selects the unweighted network input file (by using Physical Network). Next the weighted network input file is selected and stored as a list of edges (by using the Conceptual Network). Finally, by means of the Similarity File module, the user loads a file containing the mapping of the nodes of the two input networks, then sets parameter $\delta$, which represents the greatest allowed distance on the physical network. A value of $\delta$ equal to 1 means that the nodes in the physical network must be adjacent [18].

Experiments were conducted measuring the time needed to analyse modular communities, considering a set of dual input networks with a growing number of both nodes and edges. Table 1 reports execution time $T_{all}$ measured by summing three values: (*i*) the $T_{load}$, indicating the network loading time; (*ii*) $T_{align}$ which is the time required to calculate the weighted alignment graph; (*iii*) one of the two values $T_{dcs}$ or $T_{com}$ indicating the time used to analyse the communities.

In the following we report the experiments performed on the networks with characteristics and parameters.

### Analysis of communities on synthetic networks

We built 100 synthetic DNs, each one containing 200 communities ($Com_{kn,i}$, with *i* varying from 1 to 200). For each DN in this experiment we have a physical network with 500 nodes and 3000 edges and a conceptual network with 500 nodes and 4000 edges (see Table 2). We generated such graphs as follows: (i) we initially built a graph with 500 nodes and 0 edges (ii) we randomly created 200 communities of different sizes ranging from 4 to 100 in terms of node size. For each community nodes in the same group are connected with probability $p_{in}$ and the nodes of different groups are connected with probability $p_{out}$ [30].

We evaluate the results by comparing each *extracted* community $Com_{ex,j}$ with each known community $Com_{kn,i}$ contained in one of the synthetic DN. We evaluate performances for communities by using a sensitivity value called *Com sensitivity*, indicated as $Sn_{Com}$. This represents the coverage of a known community by its best-matching extracted community, i.e., the maximal fraction of nodes in the community found in a common extracted community. We also use a prediction index for communities, called *Com-wise Positive Predictive Value*, indicated as $PPV_{Com}$, which represents how well an extracted community is able to predict its best-matching in the known (i.e. real) community. The $PPV_{i,j}$ is the proportion of the members of a detected community belonging to the true community *i*, with respect to the total number of the members of this community assigned to all true community. Formally it is expressed as:

1 $$\begin{aligned} PPV_{i,j}=\frac{C{i,j}}{\sum \limits _{i=1}^{n}{C_{i,j}} } \end{aligned}$$

To characterise the PPV of a whole experiment of community detection we compute a *Com-wise Positive Predictive Value*, indicated as $PPV_{Com}$ as the weighted average of all the $PPV_{i,j}$.

Finally, in order to estimate the overall correspondence between a result (i.e., a set of extracted modular communities) and the collection of known modular communities, we evaluate the weighted means of all *PPV* values (averaged over all extracted communities) and $Sn_{Com}$ values (averaged over all known communities). The resulting statistics, clustering-wise *PPV* and clustering-wise *Sn*, provide information on the quality. We integrate the two measures by computing the geometrical accuracy ($Acc_{Com}$), defined as the average geometrical mean of *Sn* and *PPV*.

The results are reported in Tables 3 and 4 which summarise the performances of the use of Dual Network Analyser versus the Louvain algorithm used on the conceptual network only. Results are measured by using the average values evaluated on the runs over each of the 100 networks for the considered measures. As a final result, Dual Network Analyser ran over almost 100 networks outperforming the results obtained by using the Louvain algorithm only.

### Analysis of modular communities from biological dual networks

To show how the Dual Network Analyser is able to analyse modular communities in the biological domain, we built a biological DN containing protein information. We considered both the physical and conceptual interactions of proteins. Using the STRING database [47], which contains functional associations of proteins, we built a conceptual network of protein interactions. We also used the I2D [48] database containing data related to protein-to-protein physical interactions to build the physical network. To summarise, two networks containing protein information were built as follows:

- a conceptual network, which represents the association’s strength accross a group of proteins contained in the STRING database;
- a physical network, which stores the binary interactions existing in the I2D database of proteins belonging to the previous group.

We used Dual Network Analyser to analyse communities from the two networks containing 19, 354 nodes and 5, 879, 727 edges. We performed tests by using different $\delta$ values to obtain the better performance. We then set a $\delta$ parameter value equal to 4, and obtained 25 top modular communities. The use of Dual Network Analyser in this scenario is reported in Fig. 8.

The analysis of the input DN led us to 18 communities. The biggest community contains 176 edges. We performed a biological enrichment for each community to test their biological relevance by tuning the $p$-value for multiple test and using the DAVID platform. The result was that all of the communities found were biologically significant. Considering the biggest community by using $p$-value < 0.05 (resulting of multiple hypothesis corrections) we found the following enriched terms: (*i*) GO:0045955 negative regulation of calcium ion-dependent exocytosis; (*ii*) GO:0090314 positive regulation of protein which targeting to membrane; (*iii*) GO:1900078 positive regulation of cellular response to insulin stimulus; (*iv*) GO:1904707 positive regulation of vascular smooth muscle cell proliferation. These results suggest that the proteins in the biggest community may interact (directly) since they share a set of common functions.

### Analysing modular communities from social networks

We performed additional tests using the Dual Network Analyser on social networks and a GoWalla dataset. GoWalla is a social network used to share the location of users who share their positions with friends after logging into the social network [49]. User information, their positions and their friendships are available as a part of the SNAP datasets collection [50].

GoWalla dataset may be represented as a Dual Network as follows. A physical network can represent the friendship network, where each node is a user and each pair of users who happen to be friends are connected by an unweighted edge. In the examined case, the whole physical network consists of 196, 591 nodes and 950, 327 edges. Each user has a list of positions associated for every time he/she logged in the system (i.e., indicated as *check-in* in the GoWalla web site). We calculated the distances between the users expressed as distance among check-ins. In the case of multiple check-ins, we considered the average of all the check-ins. We then normalised all the distances with respect to the maximum distance of all the users. Therefore, nodes representing users who are close to each other, will be connected by edges weighting close to one, while weights close to zero indicate distant users. Two geographically close users might not be friends, whereas two friends may be geographically far apart. A *Community* in this case represents a set of users connected in a friendship network. Analysing only the conceptual network therefore, may result in missing all the information on friendships.

By analysing the DN from GoWalla dataset we found a total of 26 communities. If the biggest community found by our tool is considered, we obtain 175 related GoWalla users. From this set, only 100 users have mutual friendship. The remaining 75 users can be considered as a positive result since it contains information about new friendships.

## Discussion

In this section we report the numerical simulations and numbers obtained by using the proposed method. The proposed method is able to identify modular communities as proof of principle. We demonstrate that our findings are better than other aforementioned classical approaches by directly applying the Louvain algorithm to DNs. The quality is evaluated in two ways: (*i*) we first show the ability of our approach to recover known modular and then (*ii*) we show that our solutions are better than those of other methods.

In the case of synthetic data, we generated 100 test DNs representing communities. The results quality have been evaluated by comparing each found community with each known containing community. Sensitivity is evaluated, proving quality in terms of efficacy. The Louvain algorithm is applied to conceptual networks sub-graphs which are then induced on the physical networks. Thus, we reduced the cluster on the conceptual network to find a connected sub-graph on the physical one. Table 3 summarises the performance of the method measured by using the average value evaluated on the runs over each of the 100 networks also for normalised Mutual Information (MI), Rand index and F-score. F-score is defined as:

2 $$\begin{aligned} F-score=\frac{ 2*Precision*Recall}{Precision+Recall} \end{aligned}$$

where

3 $$\begin{aligned} Precision=\frac{ validCommunities \cap AllCommunities}{validCommunities} \end{aligned}$$

and

4 $$\begin{aligned} Recall =\frac{validCommunities \cap AllCommunities}{AllCommunities} \end{aligned}$$

indicates, respectively, the number of *valid* found communities with resepct to all those found, whereas recall indicates the number of found communities with respect to all possible ones. The final result of our method averaged over 100 networks outperforms the use the Louvain algorithm alone.

Similarly, we presented the use of Dual Network Analyser on biological networks as well as social networks. The latter is focused on relating connections, friendship and geographical positions by using the GoWalla dataset. The biological network focuses on protein-to-protein interactions. Both examples are mapped onto conceptual and physical networks by using the Dual Network Analyser to identify communities. Dual Network Analyser found communities from datasets from GoWalla suggesting new friendships. Also, communities of proteins with interesting functions have been extracted by running Dual Network Analyser on protein-to-protein interactions relations. The proposed tool has also been measured in terms of results.

## Conclusions

We presented a tool to extract communities from DNs. We considered DNs as composed of pairs of graphs: an unweighted one (physical network) and an edge-weighted one (conceptual network). The tool called Dual Network Analyser has been tested on real and synthetic datasets, demonstrating the effectiveness of our approach in analysing relevant measures from DNs efficiently. The tool, presenting a user-friendly GUI, is available online.

## Availability and requirements

- Project name: DN-Analyser
- Project home page: https://github.com/hguzzi/DNANALYZER
- Operating system(s): Platform independent
- Programming language: Python 3
- Other requirements: Python 3.7 or higher, NetworkX, TKinter, Netwulf, cdlib
- License: GNU GPL
- Any restrictions to use by non-academics: Non Commercial Use Only, CC-BY

## Data Availability

GoWalla Dataset: http://snap.stanford.edu

## Abbreviations

DN: Dual Networks
DCS: Densest Connected Subgraph

## Footnotes

1 https://codeocean.com/capsule/7601009/tree

2 https://cdlib.readthedocs.io/en/latest/

3 https://codeocean.com/capsule/7601009/tree
